# Audit on Drug Screening Practice on Inpatient Psychiatric Wards

**DOI:** 10.1192/bjo.2025.10598

**Published:** 2025-06-20

**Authors:** Mohamed Hamid, Mohamed Ahmed, Mythreyi Shekar

**Affiliations:** Leicestershire Partnership NHS Trust, Leicester, United Kingdom

## Abstract

**Aims:** The relationship between mental illness and substance misuse is well established. Early identification through drug testing can inform more holistic management plans. This audit aims to check the compliance of the current practice on acute psychiatric wards with the Trust policy for drug screening, it also aims to draw conclusions, and recommend changes to increase the compliance and benefits from implementing the policy.

**Methods:** Data was collected retrospectively from two adult acute psychiatric wards, including a sample of 20 male and 20 female patients admitted in 2024.

The parameters assessed were:

The presence of any documentation regarding drug testing on admission.

If the drug test was offered, accepted or refused, and if the results were documented.

If the positive results were acted on, such as referrals to substance misuse services.

**Results:** Any documentation related to drug screening was present in 23 out of 40 patient records (57.5%).

This indicates that nearly half of the patients admitted lacked proper documentation of whether a drug test was indicated, considered, offered, or completed.

21 out of 40 patients (52.5%) were offered a drug test.

In 4 cases, drug screening was recommended as part of the plan but was not offered or followed through. Reasons for this were not recorded.

Among the 21 tests offered, 15 patients (71.4%) completed the test. 8 (53.3%) were positive and 7 (46.7%) were negative.

6 patients (28.6%) refused UDS, but the reasons for refusal were not documented.

5 out of 8 patients with positive drug test results were referred to the substance misuse service.

**Conclusion:** This audit highlights inconsistencies in drug testing practices on inpatient wards, particularly regarding documentation, offering of tests, and follow-up on the results.

Recommended changes are as follows:

Drug screening should be offered to all inpatient groups, results should be acted on appropriately.

Improving documentation: The inpatient teams to ensure documenting if drug testing has been or should be offered, if it was accepted or refused, its results, and if positive, the follow-up plans.

By implementing those changes, drug testing can become a more effective tool for identifying and managing substance misuse, ultimately improving patient outcomes.

Findings and recommendations for change are being circulated in the Trust, and a re-audit following the implementation of recommendations will be undertaken after 3 months to evaluate the effectiveness of changes and ensure continuous improvement.

